# Nitrogen transfer and cross-feeding between *Azotobacter chroococcum* and *Paracoccus aminovorans* promotes pyrene degradation

**DOI:** 10.1038/s41396-023-01522-w

**Published:** 2023-09-29

**Authors:** Xia Wang, Ying Teng, Xiaomi Wang, Yongfeng Xu, Ran Li, Yi Sun, Shixiang Dai, Wenbo Hu, Hongzhe Wang, Yanning Li, Yan Fang, Yongming Luo

**Affiliations:** 1 Key Laboratory of Soil Environment and Pollution Remediation, Institute of Soil Science, Chinese Academy of Sciences, Nanjing, 210008 China; 2 University of the Chinese Academy of Sciences, 100049 Beijing, China; 3 Institute of Geology and Palaeontology, Chinese Academy of Sciences, Nanjing, 210008 China

**Keywords:** Soil microbiology, Transcriptomics

## Abstract

Nitrogen is a limiting nutrient for degraders function in hydrocarbon-contaminated environments. Biological nitrogen fixation by diazotrophs is a natural solution for supplying bioavailable nitrogen. Here, we determined whether the diazotroph *Azotobacter chroococcum* HN can provide nitrogen to the polycyclic aromatic hydrocarbon-degrading bacterium *Paracoccus aminovorans* HPD-2 and further explored the synergistic interactions that facilitate pyrene degradation in nitrogen-deprived environments. We found that *A. chroococcum* HN and *P. aminovorans* HPD-2 grew and degraded pyrene more quickly in co-culture than in monoculture. Surface-enhanced Raman spectroscopy combined with ^15^N stable isotope probing (SERS − ^15^N SIP) demonstrated that *A. chroococcum* HN provided nitrogen to *P. aminovorans* HPD-2. Metabolite analysis and feeding experiments confirmed that cross-feeding occurred between *A. chroococcum* HN and *P. aminovorans* HPD-2 during pyrene degradation. Transcriptomic and metabolomic analyses further revealed that co-culture significantly upregulated key pathways such as nitrogen fixation, aromatic compound degradation, protein export, and the TCA cycle in *A. chroococcum* HN and quorum sensing, aromatic compound degradation and ABC transporters in *P. aminovorans* HPD-2. Phenotypic and fluorescence in situ hybridization (FISH) assays demonstrated that *A. chroococcum* HN produced large amounts of biofilm and was located at the bottom of the biofilm in co-culture, whereas *P. aminovorans* HPD-2 attached to the surface layer and formed a bridge-like structure with *A. chroococcum* HN. This study demonstrates that distinct syntrophic interactions occur between *A. chroococcum* HN and *P. aminovorans* HPD-2 and provides support for their combined use in organic pollutant degradation in nitrogen-deprived environments.

## Introduction

Microbial remediation is a promising nature-based, sustainable, and green remediation strategy [1, 2]. Nitrogen availability is vital for maintaining bioremediation processes [3, 4]. In hydrocarbon-contaminated soils (e.g., petroleum-contaminated soils), carbon availability increases, but nitrogen availability decreases dramatically, which restricts pollutant degradation by bacteria [4, 5]. Biological nitrogen fixation by diazotrophs, which convert atmospheric nitrogen to ammonia, is a major source of bioavailable nitrogen and plays a key role in maintaining elemental biogeochemical cycles and global ecosystem productivity, particularly in nitrogen-limited environments [6–8]. Hydrocarbon-degrading bacteria and diazotrophs often share the same microhabitat and coexist as a community, and these communities may be the best bioresources for remediating hydrocarbon-contaminated natural environments [9]. However, the interactions between diazotrophs and hydrocarbon-degrading bacteria in nitrogen-deficient contaminated environments have not been characterized.

The members of microbial communities may interact bidirectionally or unidirectionally in location and nutrient utilization, resulting in cooperation or competition [10, 11]. Under nutrient-limited conditions, auxotrophic bacteria rely on other members of the community to provide essential nutrients, prompting greater local exchange among cooperative bacteria and potentially increasing reciprocity [12, 13]. Consequently, in nitrogen-deficient environments, diazotrophs and degraders may interact synergistically in the degradation of organic pollutants. For instance, introducing diazotrophs into a consortium of bacterial degraders increased the degradation rate of diesel by 3.4–6.9% comparable to the increases achieved by supplementing the consortium with inorganic nitrogen [14]. The same stimulation effect was found in one study with the dechlorination rate of bacterial consortia increasing by 16% through introducing diazotrophs [15]. Another study found that stimulating diazotrophs by adding molybdenum and tungsten increased the removal rate of polycyclic aromatic hydrocarbons (PAHs) in farmland soil by 7.5% [16]. Hence, diazotrophs may indirectly contribute to the degradation of organic pollutants by providing nitrogen to degraders. However, whether diazotrophs provide nitrogen to degraders during organic pollutant degradation and the mechanism of any synergistic interactions remain unclear.

Interactions between members in different communities are frequently driven by metabolism. Resource sharing via cross-feeding of metabolites can underlie synergistic interactions between species [17, 18]. Under nutrient-limited conditions, oligotrophic strains rely on small peptides or vitamins produced by other members of the consortium for better growth and maintenance of function [19]. Bidirectional cross-feeding studies have confirmed that the division of labor between species in metabolic pathways such as amino acid synthesis, sugar synthesis, and organic pollutant degradation reduces energy costs and improves metabolic efficiency [20–22]. Some diazotrophs possess degradation function genes and can reduce environmental toxicity through aerobic degradation and anaerobic dechlorination [23, 24]. Thus, in addition to supplying nitrogen to their neighbors in contaminated nitrogen-free microhabitats, diazotrophs may also be directly involved in the degradation and bidirectional feeding of organic pollutant degradation intermediates with other degraders. Synthetic communities (SynCom) are advantageous for studying species interactions and their contributions to community function [25, 26]. The molecular and metabolic mechanisms underlying interspecies interactions in SynCom can be explored by biofilm imaging and omics analyses, such as transcriptomics and metabolomics [27–29]. Applying these methods to elucidate the molecular and metabolic mechanisms of interactions between diazotrophs and degraders in nitrogen-deficient environments contaminated with organic pollutants could enhance the efficiency of bioremediation in natural environments.

In the present study, a stable SynCom was constructed using *Paracoccus aminovorans* HPD-2, a degrader of high-molecular-weight-polycyclic aromatic hydrocarbons (HMW-PAHs) that was isolated from historically PAH-contaminated soil, and the diazotroph *Azotobacter chroococcum* HN. HMW-PAHs are a huge international environmental problem due to their persistence and recalcitrance to degradation [30, 31]. Using the HMW-PAH pyrene as the target contaminant, we determined whether *A. chroococcum* HN provides nitrogen to *P. aminovorans* HPD-2 in a contaminated nitrogen-free microhabitat using surface-enhanced Raman spectroscopy coupled with ^15^N stable isotope probing (SERS-^15^N SIP) experiments. We performed transcriptomic and metabolomic analyses of the two strains to compare their gene expression and metabolite in co-culture and monoculture in mineral salt medium (MSM). We also analyzed the external morphology and spatial three-dimensional structure of the cells in co-cultured biofilms by electron microscopy scanning and fluorescence in situ hybridization to assess cell-cell interactions.

## Materials and methods

### Strains and media

*P. aminovorans* HPD-2 was obtained by isolation and screening from historically PAH-contaminated soil collected from Wuxi, Jiangsu Province, China. *P. aminovorans* HPD-2 is an oligotrophic-nitrogen bacterium that grows slowly in nitrogen-free media and cannot fix nitrogen [32] (Fig. S1 and Supplementary Table 1). *A. chroococcum* ATCC 9043 (HN) was purchased from China General Microbiological Culture Collection Center (CGMCC). Nitrogen-free modified MSM was used for pyrene-degradation experiments [23, 33]. The medium contained the following components (L^−1^): 2 g of mannitol, 0.15 g of anhydrous CaCl_2_, 0.2 g of MgSO_4_ 7H_2_O, 0.04 g of FeSO_4_ 7H_2_O, 0.005 g of NaMoO_4_, 0.8 g of K_2_HPO_4_, pH 6.8–7.0, and 10 mL of trace metal salt solution. The trace element solution contained (L^−1^) 10 mg of ZnSO_4_ 7H_2_O, 5 mg of MnSO_4_ H_2_O, 330 mg of H_3_BO, 24 mg of CoSO_4_ 7H_2_O, and 5 mg of CuSO_4_ 5H_2_O, pH 7.2. *P. aminovorans* HPD-2 was cultivated in Luria-Bertani (LB) medium containing (L^–1^) 10 g of NaCl, 5 g of yeast extract, and 10 g of tryptone. *A. chroococcum* HN was cultivated in Brown’s medium [33] containing (L^−1^) 5 g of glucose, 0.15 g of anhydrous CaCl_2_, 0.2 g of MgSO_4_ 7H_2_O, 0.04 g of FeSO_4_ 7H_2_O, 0.005 g of NaMoO_4_, and 0.8 g of K_2_HPO_4_, pH 6.8–7.0.

### Monoculture and interspecies inhibition test

Colonies of *P. aminovorans* HPD-2 and *A. chroococcum* HN were inoculated into 250 mL of LB or Brown’s medium, respectively, and incubated in an orbital shaker at 150 rpm and 30 °C for 24 h. The cells were separated from the medium by centrifugation, washed three times with MSM and re-suspended in MSM to obtain a bacterial suspension. The optical density at a wavelength of 600 nm (OD_600_) of the bacterial suspension was measured (Bio-TekμQuant, Winooski, VT) and adjusted to OD_600_ value of 1.0 (2.8 ×10^8^ CFU mL^–1^).

Antagonism experiments between *P. aminovorans* HPD-2 and *A. chroococcum* HN were performed in MSM agar plates by the punch hole method [34]. Briefly, MSM plates supplemented with pyrene (1 mg L^−1^) were perforated and inoculated with strains in two ways. On one side of the plate, equal volumes of monoculture of *P. aminovorans* HPD-2 and *A. chroococcum* HN were inoculated in separate holes and in the same hole. On the other side of the plate, pairs of holes with different distances between holes were inoculated separately with equal volumes of monoculture of *P. aminovorans* HPD-2 and *A. chroococcum* HN. All plates were incubated at 30 °C for 72 h.

### Growth and pyrene degradation during bacterial cultivation

A volume of pyrene stock solution was added to a 50-mL conical flask containing 10 mL of MSM to obtain a final pyrene concentration of 1 mg L^−1^. Next, suspensions of *P. aminovorans* HPD-2 and/or *A. chroococcum* HN at OD_600_ 1.0 were added to the conical flask as a monoculture or co-culture (volume, 1:1) as a 5% inoculum. The non-inoculated treatment group was a blank control. All cultures were set up in three replicates. The conical flasks were placed in a shaker (30 °C, 150 rpm) for 10 days. On days 0, 1, 3, 5, 7, and 10, samples were removed for destructive sampling to extract pyrene and pyrene degradation intermediates. The OD_600_ of the sample was measured to characterize the bacterial growth. The growth rate of live bacteria (colony-forming units, CFU) was determined by dilution plating (LB medium and Brown’s medium) and colony counting.

### Detection of pH and ammonium ions (NH_4_^+^) during bacterial cultivation

The pH of the cultures was determined using a pH meter (TOA, Co. Ltd.). The concentration of NH_4_^+^ released during pyrene degradation was determined by ion chromatography [35]. Briefly, the culture supernatant was filtered through a 0.22-μm aqueous-phase microporous membrane to remove impurities and injected into a Dionex ICS-1100 ion chromatograph (Dionex Corp., Sunnyvale, CA, USA). The concentration of NH_4_^+^ was calculated using the external standard method.

### Detection of pyrene and its degradation intermediates during bacterial cultivation

Pyrene was extracted from bacterial cultures by adding 2 times volumes of ethyl acetate and 0.4 g of (NH_4_)_2_SO_4_ (emulsion breaker) to the conical flask [36, 37]. The extraction was vortexed for 10 min and left to stand for 1 h. One milliliter of the upper organic phase was dried under a stream of N_2_ and re-dissolved in 1 mL of acetonitrile. The concentration of pyrene was measured by high-performance liquid chromatography (Shimadzu, Kyoto, Japan) according to a previous study [38].

Pyrene degradation intermediates were extracted and measured as described previously [36, 37]. In brief, the culture supernatant was acidified to pH 2.0 using 1 M HCl. A volume of ethyl acetate equal to the volume of the culture supernatant was added, and the organic-phase extracts were transferred to tubes and dried under a stream of N_2_. Finally, 0.1 mL of ethyl acetate and 0.1 mL of the derivatization reagent BSTFA-TMCS (99:1, v/v) were added to the tubes and reacted at 60 °C for 30 min, and the volume was adjusted to 1 mL with ethyl acetate. The solution was analyzed using Agilent gas chromatography-mass spectrometer (GC − MS, 6890A-5973N, Agilent Technologies, Wilmington, DE) equipped with an HP-5 MS capillary column (30 m × 0.25 mm × 0.25 μm). For qualitative determination, the MS system was operated in full-scan mode at m/z 50–450. The mode was electron impact energy mode, and the electron impact energy was 70 eV. The carrier gas was high-purity nitrogen, and the inlet was operated at a constant flow rate of 1.0 mL min^−1^. The injection port temperature was 280 °C. The sample volume was 1 μL without splitting, and the sample was purged after 0.75 min.

### Cross-feeding experiments

A stock solution of phthalic acid or 2,5-dihydroxybenzoic acid was added to a 50-mL conical flask containing 10 mL of MSM to achieve a final concentration of 600 μg L^−1^. A suspension of *P. aminovorans* HPD-2 or *A. chroococcum* HN at OD_600_ 1.0 was added to the conical flask as a 5% inoculum. The non-inoculated treatment group was a blank control. All cultures were set up in three replicates. The conical flasks were placed in a shaker (30 °C, 150 rpm) for 5 days, and samples were removed on days 0, 1, 3, and 5 for destructive analysis of phthalic acid or 2,5-dihydroxybenzoic acid. In addition, the OD_600_ of the culture was measured to characterize the bacterial growth potential.

Phthalic acid and 2,5-dihydroxybenzoic acid were extracted and measured according to the literature with some modifications [39, 40]. Culture samples (1 mL) were removed from the conical flasks and placed in 5-mL centrifuge tubes, and 1 mL of methanol (to precipitate the salt component) was added and mixed well with a vortexer. The tubes were centrifuged for 5 min at 5000 rpm. The supernatant was filtered through a 0.22-μm sterile organic membrane and transferred to an injection vial. The concentration of phthalic acid or 2,5-dihydroxybenzoic acid was determined by high-performance liquid chromatography-mass spectrometry (HPLC-MS, Tq8050, Shimadzu, Kyoto, Japan) on an Agilent SB-Aq column (3 mm × 150 mm, 2.7 μm, Waters) at a flow rate of 0.3 mL min^−1^ at 30 °C. The mobile phase consisted of (A) 0.08% formic acid and 1.2 mM ammonium acetate in water and (B) methanol. The sample injection volume was 5 μL, and the sample injector was maintained at 15 °C throughout the analysis. Mass spectrometry analysis was performed on an ABI/SCIEX 4500 Triple Quad™ with a Turbo V ion source. Phthalic acid and 2,5-dihydroxybenzoic acid were detected in negative ion mode. The nebulizer and heater gas pressures were 50 and 60 psi, respectively. The electrospray voltage was 5000 V, and the turbo ion spray source temperature was 500 °C.

### Bacterial cultivation and single-cell SERS experiment

Sterile MSM (5 mL) was added to a 30-mL crimp-top vial and spiked with pyrene stock solution to achieve an initial pyrene concentration of 1 mg L^–1^. Bacterial suspensions of *P. aminovorans* HPD-2 and *A. chroococcum* HN at OD_600_ 1.0 were added to the vial either alone or simultaneously (volume, 1:1) as a 5% inoculum, and the vial was sealed. The air in the headspace was replaced with ^15^N_2_ (99 atom %, purity >98.5%, Aladdin, China) and oxygen in a volume ratio of 4:1. The control treatments were supplied with lab air. All vials were placed in a shaker at 150 rpm and 30 °C in the dark for 5 days. The bacteria were harvested and washed twice with ultrapure water by centrifugation at 5000 rpm for 5 min. To prepare single-cell surface-enhanced Raman spectroscopy (SERS) samples, the cell pellet was mixed with 10 μL of concentrated Ag (∼7000 mg L^−1^) [41], and 2 μL of the mixture was dropped on an aluminum slide and air-dried prior to spectroscopy.

A scanning electron microscope (SEM) coupled with a confocal Raman microscope (TESCAN-MAIA 3 GMU, WITec, Czechia) was used for Raman spectroscopy of single cells in co-culture. First, a back-scattered electron (BSE) detector was employed to distinguish and obtain single-cell images of the two species. In order to distinguish between the two types of spherical cells in the Raman spectroscopy, then, the specimen stage from the SEM was moved to the Raman microscope inside the chamber to find the view and map it with the SEM single-cell images to eventually achieve overlap. Eventually, Raman spectra were acquired for two types of cells based on their morphology.

Raman analysis was conducted as follows. Single cells of *P. aminovorans* HPD-2 or *A. chroococcum* HN were excited by a 532-nm laser via a 100×/0.75 objective lens at a laser power of 20 μW with a grating of 600 g mm^−1^ [41]. The signal was acquired for 3 s per cell.

### Scanning electron microscopy and fluorescence in situ hybridization

Biofilm contents were measured in 96-well plates using the crystal violet staining method [42]. Biofilms for SEM and FISH experiments were prepared as follows. A sterile 18-mm circular coverslip was placed on the bottom of a 35-mm glass culture plate, and 3 mL of MSM and 6 μL of pyrene solution (final concentration of 1 mg L^−1^) were added. Next, suspensions of *P. aminovorans* HPD-2 and *A. chroococcum* at OD_600_ 1.0 were added to the culture plate alone or simultaneously (volume, 1:1) as a 5% inoculum. The biofilms on the coverslips were harvested after 7 days of incubation at 30 °C and 150 rpm in a constant-temperature shaker.

The biofilms of monocultures and co-culture of *P. aminovorans* HPD-2 and *A. chroococcum* were processed by referring to a previous publication [43]. Finally, the biofilms were freeze dried and gold sputter coated, and field emission SEM combined with energy-dispersive X-ray spectroscopy (FE-SEM-EDS, S-4800, Hitachi, Tokyo, Japan) was performed. FISH was performed as described previously [42]. In brief, biofilm samples were fixed with 4% paraformaldehyde for 6 h at 4 °C, washed three times with PBS, air dried, and dehydrated in 50%, 80%, and 100% ethanol for 3 min each at room temperature. Dehydrated slides can be stored at −20 °C for several months. Probes for *P. aminovorans* HPD-2 and *A. chroococcum* HN were commercially synthesized and labeled with 6-carboxyfluorescein (FAM, green) and the sulfoindocyanine dye indocarbocyanine (Cy3, red), respectively. The specimens were incubated with the fluorochrome-labeled oligonucleotide probes at a concentration of 25 ng μL^−1^ of hybridization buffer [0.9 M NaCl, 20 mM Tris/HCl (pH 7.2), 30% formamide, 0.01% (w/v) SDS] at 46 °C for 4 h. Following hybridization, the samples were quickly placed in a wash buffer [20 mM Tris/HCl (pH 7.5), 5 mM EDTA, 108 mM NaCl, and 0.01% (w/v) SDS] and incubated for 20 min at 48 °C. After washing, the samples were submerged in cold ddH_2_O for a couple of seconds and air dried completely at ambient temperature. Next, 10 μL of DAPI (diluted to 1 μg μL^−1^ with methanol) was added to the coverslips dropwise, and the samples were stained on ice for 10 min. After washing several times with water and air drying completely, the coverslips were fixed on slides and sealed around the perimeter with colorless nail polish. Images were acquired by confocal laser scanning microscopy (CLSM, LSM710, Carl Zeiss Light Microscopy, Germany) with a 40 × oil-immersion objective using the Quick-LUT (Look Up Table) function to set the pixel saturation limits.

### Transcriptomic analysis

A total of three treatment groups were subjected to transcriptomic analysis: the monocultures of *P. aminovorans* HPD-2 and *A. chroococcum* HN and the co-culture. All treatments were centrifuged at 5000 rpm for 15 min to collect cells. Total cellular RNA was extracted using TRIzol reagent according to the manufacturer’s instructions (Invitrogen, Carlsbad, CA), and RNA was quantified using a NanoDrop ND-1000 UV–Vis spectrophotometer (NanoDrop Technologies, Wilmington, DE, USA). RNA-Seq transcriptome libraries were constructed using an Illumina TruSeq RNA Sample Preparation Kit (San Diego, CA) and sequenced using the Illumina NovaSeq 6000 instrument by Majorbio Biopharm Technology Co., Ltd. (Shanghai, China). Raw RNA-Seq data (raw reads) were processed using in-house Perl scripts with FastQC software. Adapter and low-quality sequences were removed from the raw data to obtain clean RNA-Seq data. The Q20 and Q30 were counted and were more than 96.15% and 91.29%, respectively, indicating a good-quality assembly. The clean reads of each sample were sequenced against the assigned reference genome separately, and the matching rate ranged from 62.29% to 90.43%. Transcriptome sequencing results are shown in Supplementary Table 2. The read count data were normalized, and unigenes (*p*-adjust < 0.05 and |log2 fold change| ≥ 1) were identified as differentially expressed genes (DEGs) between two treatments using the DESeq2 (1.30.1). The sequences were annotated according to Gene Ontology (GO) enrichment analysis and Kyoto Encyclopedia of Genes and Genomes (KEGG) pathway enrichment analysis for functional and metabolic pathway analyses.

### Metabolomic analysis

Supernatants from 50 mL of the monocultures (control) or co-culture of *A. chroococcum* HN and *P. aminovorans* HPD-2 were collected by centrifugation at 5000 rpm for 15 min and freeze dried for 72 h. The solid sample (50 mg) was transferred to a 2-mL centrifuge tube, and a 6-mm-diameter grinding bead was added. Metabolites were extracted by adding 400 μL of extraction solution (methanol: water = 4:1 (v:v)) containing 0.02 mg mL^−1^ internal standard (L-2-chlorophenylalanine). The samples were ground with a Wonbio-96c (Shanghai Wanbo Biotechnology Co., Ltd) frozen tissue grinder for 6 min (−10 °C, 50 Hz), followed by low-temperature ultrasonic extraction for 30 min (5 °C, 40 kHz). The samples were stored at −20 °C for 30 min and centrifuged for 15 min (4 °C, 13000 g), and the supernatant was transferred to an injection vial for LC-MS/MS analysis. LC-MS/MS analysis was conducted on a Thermo UHPLC-Q Exactive HF-X system equipped with an ACQUITY HSS T3 column (100 mm × 2.1 mm i.d., 1.8 μm; Waters, USA) at Majorbio Biopharm Technology Co., Ltd. The mobile phase consisted of 0.1% formic acid in water: acetonitrile (95:5, v/v) (solvent A) and 0.1% formic acid in acetonitrile: isopropanol: water (47.5:47.5:5, v/v/v) (solvent B). Mass spectrometry data were collected on a Thermo UHPLC-Q Exactive HF-X Mass Spectrometer equipped with an electrospray ionization (ESI) source operating in positive mode and negative mode. Raw data were corrected and mapped using Progenesis QI (Waters Corporation, Milford, USA). Identified metabolites were annotated using KEGG for metabolic pathway analysis and compound classification. Metabolite differences between two groups were considered significant at *p* < 0.05 and VIP > 1.

### Statistical analysis

One-way analysis of variance (ANOVA) and Tukey’s HSD tests were performed using the “multcomp” package (4.0.4) to evaluate the significance of differences between treatments.

## Results

### Co-culturing *A. chroococcum* HN and *P. aminovorans* HPD-2 promotes mutual growth and pyrene degradation

**Fig. 1:**
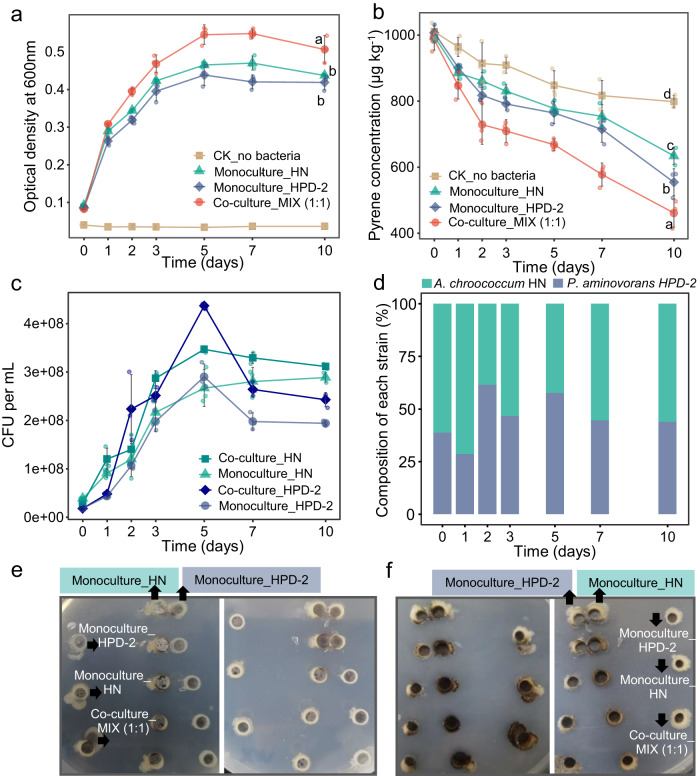
Growth and pyrene degradation curves of *A. chroococcum* HN and *P. aminovorans* HPD-2 monocultures and co-culture in nitrogen-free MSM supplemented with mannitol (0.2 g L^−1^) and pyrene (1 mg L^−1^) as carbon sources. **a** Growth curves of *A. chroococcum* HN and *P. aminovorans* HPD-2 monocultures and co-culture. **b** Pyrene degradation curves of *A. chroococcum* HN and *P. aminovorans* HPD-2 monocultures and co-culture. **c** Growth curves of *A. chroococcum* HN and *P. aminovorans* HPD-2 in monocultures and co-culture. **d** Relative proportions of *A. chroococcum* HN and *P. aminovorans* HPD-2 under co-culture conditions. Antagonism analysis between *A. chroococcum* HN and *P. aminovorans* HPD-2 after 1 day (**e**) and 3 days (**f**) of incubation in nitrogen-free MSM agar plates with mannitol (0.2 g L^−1^) and pyrene (1 mg L^−1^) as carbon sources. MIX—co-culture of *A. chroococcum* HN and *P. aminovorans* HPD-2, HN—monoculture of *A. chroococcum* HN, HPD-2—monoculture of *P. aminovorans* HPD-2. Error bars represent standard deviations (*N* = 3). Letters indicate the significance of the differences between the values of the different treatments (*p* < 0.05).

The growth characteristics and pyrene degradation of co-cultured *A. chroococcum* HN and *P. aminovorans* HPD-2 were compared with the corresponding monocultures. The co-culture and monocultures both grew rapidly after 1 day of incubation, implying that both bacteria used mannitol as the main carbon source for growth during the initial stage of culture (Fig. 1a). After 10 days of incubation, the pyrene concentration in the co-culture decreased from 985.99 μg kg^−1^ to 461.02 μg kg^−1^, resulting in a degradation rate of 32.39% after excluding the degradation in the abiotic control (20.91%) (Fig. 1b and Supplementary Table 3). By contrast, the pyrene concentration in the *A. chroococcum* HN and *P. aminovorans* HPD-2 monocultures decreased from 996.36 μg kg^−1^ and 1006.83 μg kg^−1^ to 633.95 μg kg^−1^ and 554.24 μg kg^−1^, respectively, corresponding to degradation rates of 15.45% and 24.05%. Moreover, dilution plating colony counting showed that both *A. chroococcum* HN and *P. aminovorans* HPD-2 grew better in co-culture than in monoculture (Fig. 1c). Interestingly, co-culture in MSM strongly favored the growth of *A. chroococcum* HN during the lag phase, potentially due to greater utilization of mannitol compared with *P. aminovorans* HPD-2 in the nitrogen-free environment (Fig. 1d). There was a shift toward increased *P. aminovorans* HPD-2 in the exponential phase, possibly due to the consumption of nitrogen released by *A. chroococcum* HN. Thus, co-culture not only promoted the growth of *A. chroococcum* HN and *P. aminovorans* HPD-2 but also significantly accelerated the degradation of pyrene. The antagonism experiments also visually demonstrated that *A. chroococcum* HN and *P. aminovorans* HPD-2 were better able to promote each other’s growth when co-cultured in direct contact (Fig. 1e, f).

### *A. chroococcum* HN provides nitrogen to *P. aminovorans* HPD-2 to mitigate nutrient-poor environments

**Fig. 2:**
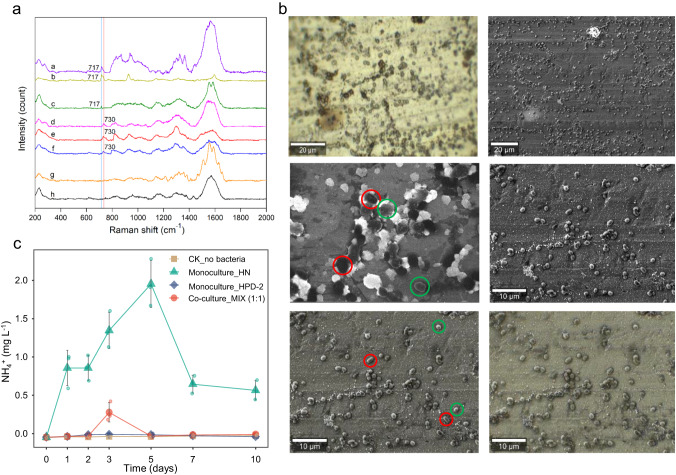
Characteristic of NH_4_^+^ concentrations and nitrogen-absorbing cell in studied cultures. **a** SERS spectra of *A. chroococcum* HN and *P. aminovorans* HPD-2 incubated with ^15^N_2_ and ^14^N_2_ in monoculture and co-culture. Spectra a, b, d, and e correspond to *A. chroococcum* HN incubated with ^15^N_2_ in co-culture, ^15^N_2_ in monoculture, ^14^N_2_ in co-culture, and ^14^N_2_ in monoculture, respectively; spectra c and f belong to *P. aminovorans* HPD-2 incubated with ^15^N_2_ or ^14^N_2_ in co-culture, respectively; and spectra g and h correspond to *P. aminovorans* HPD-2 incubated with ^15^N_2_ or ^14^N_2_ in monoculture, respectively. **b** Scanning electron microscopy mapping images combined with Raman imaging of *A. chroococcum* HN and *P. aminovorans* HPD-2 in co-culture. The red and green circles indicate cells of *A. chroococcum* HN and *P. aminovorans* HPD-2 in co-culture, respectively. **c** Changes in NH_4_^+^ concentrations in the monocultures and co-culture of *A. chroococcum* HN and *P. aminovorans* HPD-2. MIX—co-culture of *A. chroococcum* HN and *P. aminovorans* HPD-2, HN—monoculture of *A. chroococcum* HN, HPD-2—monoculture of *P. aminovorans* HPD-2. Error bars represent standard deviations (*N* = 3).

To investigate whether *A. chroococcum* HN provided nitrogen to *P. aminovorans* HPD-2 during pyrene degradation in co-culture, single-cell SERS − ^15^N SIP experiments were performed. The Raman spectra of monocultured *A. chroococcum* showed bands at 730 or 717 cm^−1^ due to N assimilation when incubated with ^15^N_2_ or ^14^N_2_ (Fig. 2a and Supplementary Fig. S2). However, the Raman spectra of monocultured *P. aminovorans* HPD-2 did not include these bands due to the inability of this bacterium to fix nitrogen. Scanning electron microscopy observations combined with Raman imaging revealed that the Raman spectra of both *A. chroococcum* HN and *P. aminovorans* HPD-2 in co-culture included bands at 730 or 717 cm^−1^ when incubated with ^15^N_2_ or ^14^N_2_, respectively (Fig. 2a, b), implying that *A. chroococcum* HN provided nitrogen for *P. aminovorans* HPD-2. Furthermore, *A. chroococcum* HN released significant amounts of NH_4_^+^ during pyrene degradation in monoculture, whereas the monoculture of *P. aminovorans* HPD-2 did not (Fig. 2c). It is worth noting that no NH_4_^+^ was detected in the co-culture of *A. chroococcum* HN and *P. aminovorans* HPD-2, suggesting that *A. chroococcum* HN provided nitrogen to *P. aminovorans* HPD-2 by releasing NH_4_^+^.

### Cross-feeding between *A. chroococcum* HN and *P. aminovorans* HPD-2 in pyrene degradation

**Fig. 3:**
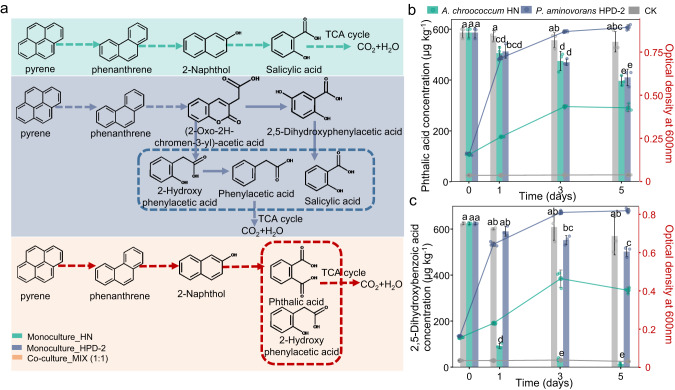
Possible pyrene degradation pathways in studied cultures. **a** Possible pyrene degradation pathways inferred from intermediate metabolites of pyrene degradation in the monocultures and co-culture of *A. chroococcum* HN and *P. aminovorans* HPD-2. The solid and dotted lines represent the paths between the lines where the two substances can or cannot be degraded in one step, respectively. MIX—co-culture of *A. chroococcum* HN and *P. aminovorans* HPD-2, HN—monoculture of *A. chroococcum* HN, HPD-2—monoculture of *P. aminovorans* HPD-2. **b** Changes in the concentrations of phthalic acid and growth curves of *A. chroococcum* HN and *P. aminovorans* HPD-2 in nitrogen-free MSM supplemented with mannitol (0.2 g L^−1^) and phthalic acid (600 μg L^−1^) as carbon sources. **c** Changes in the concentrations of 2, 5-dihydroxybenzoic acid and growth curves of *A. chroococcum* HN and *P. aminovorans* HPD-2 in nitrogen-free MSM supplemented with mannitol (0.2 g L^−1^) and 2, 5-dihydroxybenzoic acid (600 μg L^−1^) as carbon sources. Error bars represent standard deviations (*N* = 3). Letters indicate the significance of the differences between the values of the different treatments (*p* < 0.05).

Since *A. chroococcum* HN degraded pyrene, we speculated that cross-feeding of pyrene intermediate metabolites between *A. chroococcum* HN and the degrader *P. aminovorans* HPD-2 may occur during pyrene degradation. To evaluate this possibility, changes in pH and pyrene metabolites were examined in the monocultures, co-culture, and cell-free controls. In the monoculture of *A. chroococcum* HN, the pH was 7.0 on day 0 and gradually increased to approximately 7.4 as the cells grew and pyrene was degraded; the pH of the monoculture of *A. chroococcum* HN was not significantly different from that of the cell-free control (Supplementary Fig. S3). After day 7 of culture, three pyrene degradation intermediates were detected: phenanthrene, 2-naphthol, and salicylic acid (Supplementary Fig. S4 and Supplementary Table 4). The pH of the *P. aminovorans* HPD-2 monoculture was 7.3 on day 0 but rapidly decreased to 6.7 on day 1 and then gradually increased to approximately 7.0 as pyrene was degraded. The pH of the *P. aminovorans* HPD-2 monoculture was significantly lower than that of the cell-free control. Six pyrene degradation intermediates were detected in the monoculture of *P. aminovorans* HPD-2: phenanthrene, (2-oxo-2H-chromen-3-yl)-acetic acid, 2,5-dihydroxybenzoic acid, 2-hydroxyphenylacetic acid, phenylacetic acid, and salicylic acid. In the co-culture of *A. chroococcum* HN and *P. aminovorans* HPD-2, the pH increased gradually with the degradation of pyrene and was between the pH values of the two monocultures (7.0–7.2). Four pyrene degradation intermediates were produced in the co-culture: phenanthrene, 2-naphthol, 2-hydroxyphenylacetic acid, and phthalic acid (Supplementary Fig. S4 and Supplementary Table 4). Because phenanthrene was detected in all cultures, we deduced that the pathway of pyrene degradation by the bacteria was oxidative ring opening and decarboxylation of pyrene to phenanthrene by mono- or dioxygenase, followed by continued degradation via the phthalic or salicylic acid metabolic pathway (Fig. 3a).

Based on the functional genes associated with PAH metabolism in the whole genomes of the two species and the differences in pyrene degradation intermediates (Fig. 3a and Supplementary Fig. S5), 2,5-dihydroxybenzoic acid and phthalic acid feeding experiments were performed in monocultures of *P. aminovorans* HPD-2 and *A. chroococcum* HN. Both *A. chroococcum* HN and *P. aminovorans* HPD-2 grew well in the experimental system supplemented with phthalic acid. Compared with the sterile control treatment, both *A. chroococcum* HN and *P. aminovorans* HPD-2 degraded approximately 31.22% of the phthalic acid after 5 days of culture (Fig. 3b). In the experimental system with 2,5-dihydroxybenzoic acid, both *A. chroococcum* HN and *P. aminovorans* HPD-2 grew well. However, compared to the sterile control treatment, *A. chroococcum* HN reduced the concentration of 2,5-dihydroxybenzoic acid from 624.25 μg kg^−1^ to approximately 92.21 μg kg^−1^ after 1 day of incubation, corresponding to a degradation rate of 85.23% (Fig. 3c). For *P. aminovorans* HPD-2, the degradation rate was only 19.56% after 5 days of incubation. These results suggest that cross-feeding of 2,5-dihydroxybenzoic acid or phthalic acid occurred between *P. aminovorans* HPD-2 and *A. chroococcum* during pyrene degradation.

### Transcriptomics and metabolomics of *A. chroococcum* HN and *P. aminovorans* HPD-2 in co-culture

**Fig. 4:**
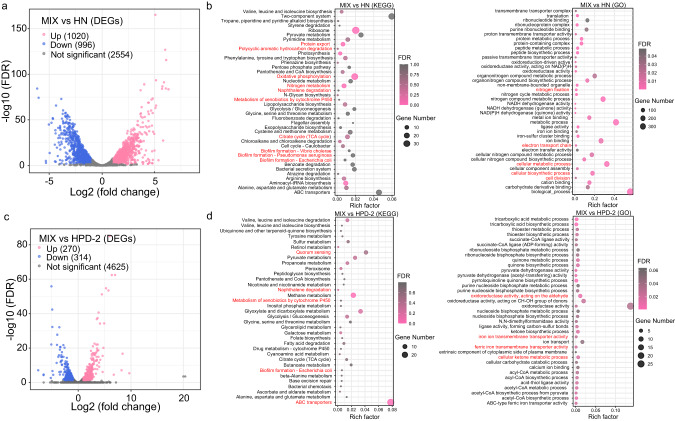
Volcano plots of the differentially expressed genes and enrichment analysis of KEGG and GO functional categories in *A. chroococcum* HN and *P. aminovorans* HPD-2 in the co-culture compared with the corresponding monocultures. **a** Volcano plots of differentially expressed genes in *A. chroococcum* HN in co-culture versus monoculture. **b** Enrichment analysis of KEGG and GO functional categories in *A. chroococcum* HN in co-culture versus monoculture. **c** Volcano plots of the differentially expressed genes in *P. aminovorans* HPD-2 in co-culture versus monoculture. **d** Enrichment analysis of KEGG and GO functional categories in *P. aminovorans* HPD-2 in co-culture versus monoculture. MIX—co-culture of *A. chroococcum* HN and *P. aminovorans* HPD-2, HN—monoculture of *A. chroococcum* HN, HPD-2—monoculture of *P. aminovorans* HPD-2. Three replicates (*N* = 3) were used for transcriptomics analyses.

To uncover the molecular mechanisms of the interactions between *A. chroococcum* HN and *P. aminovorans* HPD-2 for pyrene degradation in co-culture, we performed transcriptomics and extracellular untargeted metabolomics on the monocultures and co-culture. After standardizing RNA read counts, 1020 genes were significantly upregulated and 996 genes were significantly downregulated in *A. chroococcum* HN in the co-culture compared with the monoculture (adj. *p* ≤ 0.05 and |log2FC | ≥ 1) (Fig. 4a). The differentially expressed genes (DEGs) were analyzed using KEGG ontology and GO functional category enrichment (Fig. 4b and Supplementary Fig. S6). The significantly upregulated DEGs were distributed among nitrogen metabolism, cellular metabolism, degradation of aromatic compounds, TCA cycle, amino acid metabolism, oxidative phosphorylation, biofilm formation, and protein export (Fig. 4b). In *P. aminovorans* HPD-2, 270 genes were significantly upregulated and 314 genes were significantly downregulated in the co-culture compared with the monoculture (Fig. 4c). The upregulated DEGs were mainly involved in quorum sensing, degradation of aromatic compounds, amino acid metabolism, oxidoreductase activity, cellular metabolism, ABC transporters, and iron transport (Fig. 4d).

**Fig. 5:**
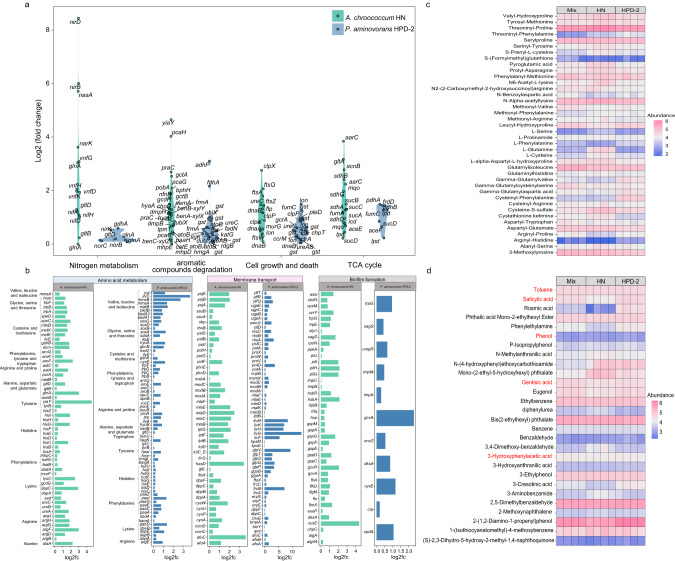
Distribution of upregulated genes and metabolites across major pathways in the co-culture compared with the monoculture of each species and identification of the main differential metabolites. **a** Genes related to nitrogen metabolism, aromatic compound degradation, cell growth and death and the TCA cycle were upregulated in *A. chroococcum* HN and *P. aminovorans* HPD-2 in co-culture. **b** Genes related to amino acid metabolism, membrane transport and biofilm formation were upregulated in *A. chroococcum* HN and *P. aminovorans* HPD-2 in co-culture. Upregulated gene expression across major pathways was measured by the log 2-fold change (Log2FC) of gene expression in each species in the co-culture compared with the corresponding monoculture. **c** Metabolite abundances of amino acids and peptides in the monocultures and co-culture of *A. chroococcum* HN and *P. aminovorans* HPD-2. **d** Metabolite abundances of aromatic compounds in the monocultures and co-culture of *A. chroococcum* HN and *P. aminovorans* HPD-2. MIX—co-culture of *A. chroococcum* HN and *P. aminovorans* HPD-2, HN—monoculture of *A. chroococcum* HN, HPD-2—monoculture of *P. aminovorans* HPD-2. Four replicates (*N* = 4) were used for metabolomics analyses.

Based on the KEGG results, the analysis further focused on upregulated DEGs associated with major metabolic pathways such as nitrogen metabolism, amino acid metabolism, membrane transport, degradation of aromatic compounds, cellular metabolism, TCA cycle, and biofilm formation in *A. chroococcum* HN and *P. aminovorans* HPD-2. Among nitrogen metabolic pathways, many upregulated DEGs in *A. chroococcum* HN belonged to nitrogen fixation pathways, including *nifH*/*D*/*K* (nitrogenase iron protein; nitrogenase molybdenum-iron protein alpha chain/subunit beta) and *vnfD*/*K*/*G* (vanadium-dependent nitrogenase alpha chain/beta chain; vanadium nitrogenase delta subunit). By contrast, few of the upregulated genes in *P. aminovorans* HPD-2 were related to nitrogen metabolism (Fig. 5a and Supplementary Table 5). In addition, a large number of genes related to amino acid synthesis and metabolism were upregulated in *A. chroococcum* HN and *P. aminovorans* HPD-2, particularly those related to amino acids such as glutamate, alanine, arginine, aspartate, glycine, serine, threonine, tryptophan, valine, leucine, isoleucine, cysteine, methionine, proline, lysine, and tyrosine (Fig. 5b and Supplementary Table 6). In the nitrogen-free co-culture environment, nitrogen fixed by *A. chroococcum* HN was the only source of nitrogen for cell growth. Consequently, the upregulation of the nitrogen fixation pathway in *A. chroococcum* HN and the amino acid synthesis pathways in both species indicate that *P. aminovorans* HPD-2 utilized nitrogen fixed by *A. chroococcum* HN, such as NH_4_^+^. The protein and peptide metabolic process and protein export pathways of *A. chroococcum* HN were also significantly upregulated (Fig. 4b), and analysis of the membrane transport pathway revealed that the main upregulated genes were *livH*/*M*/*G*/*F* (branched-chain amino acid transport system permease protein/ATP-binding protein) and *dppF*/*C*/*B*/*A* (dipeptide transport system ATP-binding protein/permease protein/substrate-binding protein) (Fig. 5b and Supplementary Table 7). The same genes were upregulated in *P. aminovorans* HPD-2. In addition, the ammonium transporter in the *P. aminovorans* HPD-2 membrane transport pathway was upregulated. We performed metabolomics analysis to compare amino acid and peptide metabolites between the co-culture and monocultures. The abundances of most dipeptides in the co-culture were significantly lower than those in the *A. chroococcum* HN monoculture but significantly higher than those in the *P. aminovorans* HPD-2 monoculture, including aspartylglutamate, prolylasparagine, serinyltyrosine, threoninylphenylalanine, and valyl-hydroxyproline (Fig. 5c and Supplementary Table 9). These differences suggest that *P. aminovorans* HPD-2 used amino acids and dipeptides secreted by *A. chroococcum* HN as a source of nitrogen and energy for growth, consistent with the transcriptomic results.

Among aromatic degradation pathway genes, co-culture significantly upregulated genes encoding key enzymes for ring-opening of PAHs in *A. chroococcum* HN, such as *catE* (catechol 2, 3-dioxygenase), *pcaH* (protocatechuate 3, 4-dioxygenase), and a series of related dehydrogenases (Fig. 5a and Supplementary Table 5). However, co-culture did not upregulate genes encoding key enzymes associated with PAH degradation in *P. aminovorans* HPD-2. A series of downstream metabolites of aromatic compounds, including salicylic acid, gentisic acid, toluene, phenol, and 3-hydroxyphenylacetic acid, were identified by metabolomics analysis of the monocultures and co-culture (Fig. 5d and Supplementary Table 10). The abundances of salicylic acid, gentisic acid, and phenol metabolites in the co-culture were significantly increased compared to those in the *A. chroococcum* HN monoculture but significantly decreased compared to those in the *P. aminovorans* HPD-2 monoculture, implying that downstream metabolites from pyrene degradation by *P. aminovorans* HPD-2 were used as carbon sources by *A. chroococcum* HN. This result was consistent with the results of the feeding experiment.

Cell growth and death genes and TCA cycle-related genes were significantly upregulated in *P. aminovorans* HPD-2 and *A. chroococcum* HN when grown in co-culture (Fig. 5a), further indicating that these species contributed significantly to each other’s growth and carbohydrate recycling. Biofilm-related genes were significantly upregulated in both species in the co-culture (Fig. 5b and Supplementary Table 8), as was the quorum-sensing pathway in *P. aminovorans* HPD-2 (Fig. 4d). These results suggest that biofilm formation as well as intercellular signaling may play an important role in interspecies interactions.

### External morphology and three-dimensional structure of cells in biofilms from *A. chroococcum* HN and *P. aminovorans* HPD-2 co-culture

**Fig. 6:**
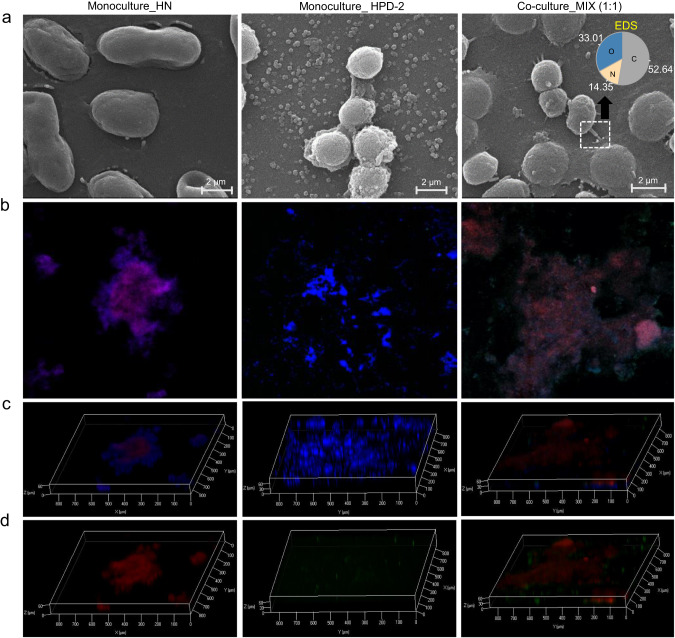
Microscopic analysis of biofilms from the *A. chroococcum* HN and *P. aminovorans* HPD-2 monocultures and co-culture. MIX—co-culture of *A. chroococcum* HN and *P. aminovorans* HPD-2, HN—monoculture of *A. chroococcum* HN, HPD-2—monoculture of *P. aminovorans* HPD-2. **a** Scanning electron micrographs of biofilms from the monocultures and co-culture and EDS analysis of the bridge-like structure observed in the co-culture biofilm. **b** Confocal laser scanning image of FISH-stained biofilm planes of *A. chroococcum* HN and *P. aminovorans* HPD-2 from the monocultures and co-culture. Images from left to right include the monoculture of *A. chroococcum* HN, monoculture of *P. aminovorans* HPD-2, and co-cultures of *A. chroococcum* HN and *P. aminovorans* HPD-2. Red represents *A. chroococcum* HN, green represents *P. aminovorans* HPD-2, and blue represents the nucleus. **c**, **d** Confocal laser scanning image of FISH-stained biofilm 3D structure from monocultures and co-culture of *A. chroococcum* HN and *P. aminovorans* HPD-2 with or without the blue of the nucleus.

To further study the interactions and interdependence of *P. aminovorans* HPD-2 and *A. chroococcum* HN in pyrene degradation, the external morphology, and three-dimensional structure of cells in biofilms from the co-culture and monocultures and the distribution of pyrene on the bacterial biofilms were examined. *A. chroococcum* HN produced a large number of biofilms along with pyrene degradation in monoculture, and electron microscopy revealed that the *A. chroococcum* HN cells were spherical and surrounded by a thick extracellular matrix (ECM), with a small amount of granular pyrene distributed on the surface (Fig. 6a and Supplementary Fig. S7). By contrast, *P. aminovorans* HPD-2 produced a small amount of biofilms. Electron microscopy of these biofilms showed that the cells were also spherical but were covered only by a thin ECM, and a small amount of granular pyrene was detected on the surface. The co-culture produced a moderate number of biofilms, and electron microscopy showed that the cells were embedded in the ECM and that there was a bridge-like structure between the cells of *A. chroococcum* HN and *P. aminovorans* HPD-2 (Fig. 6a and Supplementary Fig. S7). This structure, which EDS indicated was mainly composed of carbon, nitrogen, and oxygen, may contribute to the transportation of substances between cells. In addition, a large distribution of pyrene was found on the surface of the biofilms of both species. Spatial localization analysis by FISH further confirmed these observations. *A. chroococcum* HN and *P. aminovorans* HPD-2 maintained a stable relative biovolume in the co-culture biofilm layer (Fig. 6b). The analysis of the three-dimensional structure of the cells in the biofilm showed that *A. chroococcum* HN predominantly occupied the bottom layer and had extremely high biomass (Fig. 6c, d). By contrast, *P. aminovorans* HPD-2 occupied the top layer and had a relatively low biovolume in the biofilm layer; however, colonization was greatly increased compared to the monoculture biofilm.

## Discussion

**Fig. 7:**
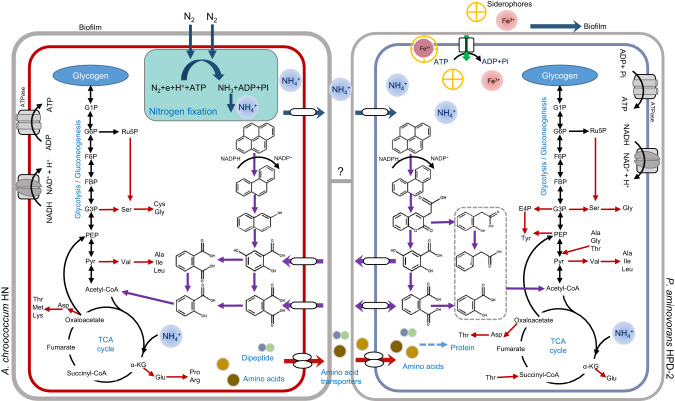
Schematic illustration of the interactions between *A. chroococcum* HN and *P. aminovorans* HPD-2 during pyrene degradation in a nitrogen-free environment. *A. chroococcum* HN produces NH_4_^+^, amino acids and peptides through biological nitrogen fixation and provides nutrients to *P. aminovorans* HPD-2 through ammonium ion and amino acid transporters. Cross-feeding of downstream intermediates of pyrene, such as 2, 5-dihydroxybenzoic acid and phthalic acid, between *P. aminovorans* HPD-2 and *A. chroococcum* HN synergistically accelerated pyrene degradation.

Nitrogen is a key nutrient limiting degraders function, and biological nitrogen fixation by diazotrophs may synergistically contribute to hydrocarbon degradation in nitrogen-free contaminated environments by providing nitrogen to degraders [4, 9]. The mechanisms of synergistic interactions that enhance function in nutrient-poor environments include cross-feeding, communication through chemical signals, and direct contact through biofilms [44–46]. In this study, we verified that nitrogen transfer, cross-feeding, and biofilm production were mechanisms of synergy between *A. chroococcum* and *P. aminovorans* for promoting growth and degradation (Fig. 7). These synergistic interactions indicate that consortia of diazotrophs and degraders would be the best bioresources for the remediation of PAH-contaminated natural environments.

Although it has been proposed that diazotrophs provide nitrogen to degraders and promote hydrocarbon degradation in different environments [5, 7], direct evidence of this relationship is limited. A study reported significant shifts in the 730 cm^–1^ SERS-^15^N SIP band for different bacterial species due to the substitution of “light” ^14^N by “heavy” ^15^N; thus, SERS-^15^N SIP is useful for identifying active nitrogen-absorbing cells [41]. In the present study, ^15^N_2_/^14^N_2_ culture experiments combined with SERS-^15^N SIP revealed that *P. aminovorans* HPD-2 was able to take up and utilize nitrogen released by *A. chroococcum* HN to mitigate nutrient-deficient environments. This study is the first to demonstrate that diazotrophs are capable of supplying nitrogen to degraders in a nitrogen-free contaminated environment.

Under nutrient-limited conditions, oligotrophic strains must rely on metabolites produced by their neighbors, such as vitamins, amino acids, and NH_4_^+^, for growth and function [20, 44, 47]. In this work, we found that *A. chroococcum* HN released large amounts of NH_4_^+^ during growth, either by secretion or by release after cell metabolic death. In general, *Azotobacter* only fixes sufficient nitrogen for its own use and cannot secrete NH_4_^+^ to other co-cultured microbes [48]. No NH_4_^+^ was detected in the co-culture and the transcriptomic data indicated that the ammonium transporter in the *P. aminovorans* HPD-2 membrane transport pathway was upregulated. This implies that the NH_4_^+^ released by *A. chroococcum* HN was utilized by *P. aminovorans* HPD-2 as a nitrogen source. The transcriptomic and metabolomic data further revealed that *P. aminovorans* HPD-2 significantly promoted the nitrogen fixation pathway of *A. chroococcum* HN in co-culture, suggesting that the nitrogen metabolites secreted by *A. chroococcum* HN cross-fed *P. aminovorans* HPD-2 and accelerated the nitrogen fixation pathway via positive feedback [49]. The up-regulation of genes related to branched-chain amino acid and dipeptide protein transport in co-cultures of *A. chroococcum* HN and *P. aminovorans* HPD-2, as well as the related metabolites identified, suggests that *A. chroococcum* HN also fed amino acids and small peptides to *P. aminovorans* HPD-2. Overall, these results further emphasize the role of synergistic interactions between microorganisms in a nutrient-deficient environment.

Co-metabolic degradation provides the benefit of removing contaminants as the degraders are not dependent on the carbon or energy source of the contaminants [50, 51]. In this study, *A. chroococcum* HN did not grow in the absence of an additional carbon source (data not shown), and 2% mannitol was added to the nitrogen-free MSM as a carbon source to promote bacterial growth. *A. chroococcum* HN degraded pyrene in monoculture, indicating its reliance on additional carbon sources for pyrene degradation. Microorganisms have diverse PAH-degrading characteristics [31]. One of the main forms of interaction between degraders in a microbial consortium during the degradation of organic pollutants such as PAHs and crude oil is the cross-feeding of metabolites [52, 53]. The degradation rate of pyrene was 1.3-2.1 times higher in the co-culture than in either monoculture, implying a synergistic effect. Metabolite analysis revealed differences in the abundances of metabolites such as 2-naphthol, phthalic acid, salicylic acid, and 2,5-dihydroxybenzoic acid between the monocultures and co-culture, further suggesting cross-feeding of pyrene metabolites between *A. chroococcum* HN and *P. aminovorans* HPD-2 in co-culture.

Genomic and metabolic data can be used to define possible interactions between partners, as has been done in recent models (constraint-based metabolic modeling) [54]. Pyrene degradation intermediates such as phthalic acid and 2,5-dihydroxybenzoic acid are the most common downstream metabolites in the degradation of PAHs and were detected in this study. Feeding experiments showed that both *A. chroococcum* HN and *P. aminovorans* HPD-2 utilized phthalic acid and 2,5-dihydroxybenzoic acid. In particular, *A. chroococcum* HN efficiently degraded 2,5-dihydroxybenzoic acid, which is produced during pyrene metabolism by *P. aminovorans* HPD-2. These results imply an effective division of labor between species with complementary genes that facilitates pollutant degradation under stress conditions through positive feedback. These positive interactions in natural soils are essential for microbial growth and survival in communities [55].

The results of transcriptome analysis showed that co-culture significantly promoted PAH degradation pathways, naphthalene degradation pathways, and xenobiotic metabolism by the cytochrome P450 pathway in *A. chroococcum* HN and *P. aminovorans* HPD-2. In particular, genes encoding key enzymes of the PAH ring-opening step, such as catechol 2, 3-dioxygenase and protocatechuate 3, 4-dioxygenase, were upregulated in *A. chroococcum* HN. In most microorganisms, the degradation of aromatic compounds begins with oxidation by dioxygenase to yield a product with two hydroxyl groups [31]. Although co-culture upregulated naphthalene degradation and xenobiotic metabolism by the cytochrome P450 pathway in *P. aminovorans* HPD-2, it did not promote the expression of key enzymes involved in PAH degradation in *P. aminovorans* HPD-2. We speculated that this occurred because as a degrader, *P. aminovorans* HPD-2 is insensitive to pyrene stress; in addition, pyrene was not the only carbon source for growth in the culture environment. By contrast, *A. chroococcum* HN relies on external carbon sources for degradation and is sensitive to pyrene stress (Fig. S8). Consequently, key PAH-degradation genes were significantly upregulated in *A. chroococcum* HN but not in *P. aminovorans* HPD-2. Furthermore, the TCA cycle, oxidative phosphorylation, and ABC transporters were all significantly upregulated in co-cultured *A. chroococcum* HN and *P. aminovorans* HPD-2, implying that these pathways are associated with pyrene degradation. The TCA cycle is a major pathway for the further degradation of PAH metabolites and for providing energy to the bacterium. Previous studies have found that the TCA cycle, ATP synthesis pathway, and ABC transporters are all upregulated when PAHs are the sole carbon source [56].

Biofilms are formed by cells adhering to each other on a substrate through the production of extracellular polymeric substance (EPS) and can act as a medium for the exchange of metabolites through cross-feeding [43, 57]. In this study, electron microscopy and FISH experiments showed that *A. chroococcum* HN produced a large number of biofilms and was found in the bottom layer of co-culture biofilms. *P. aminovorans* HPD-2 formed fewer biofilms and was tightly attached to *A. chroococcum* HN. Importantly, biofilm formation by *P. aminovorans* HPD-2 was significantly higher in the co-culture than in the monoculture, suggesting that co-culture with *A. chroococcum* HN significantly promoted *P. aminovorans* HPD-2 growth and biofilm formation. Transcriptomic data showed that co-culture promoted the biofilm formation pathways of *A. chroococcum* HN and *P. aminovorans* HPD-2, particularly the ferric ion transmembrane transporter in *P. aminovorans* HPD-2. Biofilm formation involves the regulatory function of the siderophore system, which enables iron to regulate several functional metabolites [43]. In addition, we found that co-culture significantly promoted quorum sensing in *P. aminovorans* HPD-2. Microorganisms engage in quorum sensing at the population level to enable microbial cell-to-cell communication. Quorum sensing plays a significant role in coordinating biofilm organization and regulating genes through the use of specific chemical signals known as autoinducers (AI) [58]. The control of biofilm substrate components by intracellular and extracellular signals is achieved through transcriptional and post-transcriptional regulation and environmental factors [59]. Thus, co-culture with *A. chroococcum* HN in a nitrogen-deficient environment promoted communication between *P. aminovorans* HPD-2 and *A. chroococcum* HN cells, resulting in sufficient nitrogen and division of labor to co-metabolize pyrene. Although a bridge-like structure was found between the cells of *P. aminovorans* HPD-2 and *A. chroococcum* HN, which may act as a channel for material transfer between cells [60], further in-depth investigation of biofilms is needed to determine how the cells communicate and cooperate with each other.

## Conclusions

In this study, we observed synergistic interactions between *A. chroococcum* and *P. aminovorans* during the degradation of pyrene in a nitrogen-free contaminated environment. This study is the first to show that the degrader *P. aminovorans* can receive nitrogen for growth from a syntrophic partner, *A. chroococcum*. *A. chroococcum* HN fed *P. aminovorans* HPD-2 mainly by secreting NH_4_^+^, amino acids, and peptides, thus generating a positive feedback mechanism that enhanced nitrogen fixation by *A. chroococcum* HN. Furthermore, cross-feeding of downstream metabolites of pyrene, such as 2, 5-dihydroxybenzoic acid and phthalic acid, between *P. aminovorans* HPD-2 and *A. chroococcum* HN synergistically accelerated pyrene degradation. Enhanced biofilm formation and quorum sensing by *P. aminovorans* HPD-2 may provide chemical signals and mediators for nitrogen supplementation and metabolite exchange in a nitrogen-deficient environment. In conclusion, this study provides new insights into the mechanisms of synergistic interactions between *P. aminovorans* HPD-2 and *A. chroococcum* HN in a nitrogen-deficient contaminated environment that may be useful for improving soil bioremediation.

### Supplementary information

Supporting Information for Nitrogen transfer and cross-feeding between Azotobacter chroococcum and Paracoccus aminovorans promotes pyrene degradation

Supplementary Tables

## Data Availability

Our sequences files of transcriptome are accessible from the National Center for Biotechnology Information (BioProject PRJNA995359, Sequence Read Archive accession numbers SRR25308487-SRR25308495). Raw data of GC-MS have been deposited in the Metabolights [61] (MTBLS8212, www.ebi.ac.uk/metabolights/MTBLS8212).
