# Supplementary material for: Nitrogen transfer and cross-feeding between Azotobacter chroococcum and Paracoccus aminovorans promotes pyrene degradation
Source: ISME J. 2023 Sep 29;17(12):2169–81. doi: 10.1038/s41396-023-01522-w (PMC10689768; doi:10.1038/s41396-023-01522-w)
Supplement: Supplementary file 1 — Supporting Information for Nitrogen transfer and cross-feeding between Azotobacter chroococcum and Paracoccus aminovorans promotes pyrene degradation [file 41396_2023_1522_MOESM1_ESM.docx]

**Supporting Information for**

**Nitrogen transfer and cross-feeding between *Azotobacter chroococcum* and *Paracoccus aminovorans* promotes pyrene degradation**

**Xia Wang^1,2^ ∙ Ying Teng^1,2^ ∙ Xiaomi Wang^1,2^ ∙ Yongfeng Xu^1,2^ ∙ Ran Li^1,2^ ∙ Yi Sun^1,2^ ∙ Shixiang Dai^1,2^ ∙ Wenbo Hu^1,2^ ∙ Hongzhe Wang^1,2^ ∙ Yanning Li^1,2^ ∙ Yan Fang^2,3^ ∙ Yongming Luo^1,2^**

^1^ Key Laboratory of Soil Environment and Pollution Remediation, Institute of Soil Science, Chinese Academy of Sciences, Nanjing 210008, China

^2^ University of the Chinese Academy of Sciences, Beijing 100049, China

^3^ Institute of Geology and Palaeontology, Chinese Academy of Sciences, Nanjing 210008, China

Total number of pages of Supporting Information: 15 (including cover page)

Number of Figures in Supporting Information: 8

Number of Tables in Supporting Information: 10

**Figures**

**Fig. S1** Growth of *A. chroococcum* HN and *P. aminovorans* HPD-2 in Brown medium.

**Fig. S2** Uncorrected SERS spectra of *A. chroococcum* HN and *P. aminovorans* HPD-2 incubated with ^15^N_2_ and ^14^N_2_ in monocultures and co-culture.

**Fig. S3** Change in pH value during pyrene degradation in monocultures and co-culture of *A. chroococcum* HN and *P. aminovorans* HPD-2. Error bars represent standard deviations (N = 3).

**Fig. S4** Mass spectra of the identified metabolites during pyrene degradation in monocultures and co-culture of *A. chroococcum* HN and *P. aminovorans* HPD-2.

**Fig. S5** Functional genes associated with the metabolism of PAHs in the whole genomes of *A. chroococcum* HN (a) and *P. aminovorans* HPD-2 (b). Information on *A. chroococcum* HN was obtained through RAST Server annotation of raw sequence from the National Center for Biotechnology Information. The purple, pink and blue boxes indicate that the enzymes encoded by the genes in the *P. aminovorans* HPD-2 and *A. chroococcum* HN genomes share some similarities with enzymes in metabolic pathways.

**Fig. S6 a.** Analysis of KEEG and GO functional categories down-regulation for *A. chroococcum* HN in the co-culture versus its monoculture. **b.** Analysis of KEEG and GO functional categories down-regulation for *P. aminovorans* HPD-2 in the co-culture versus its monoculture.

**Fig. S7 a.** Change in biomass of biofilms during pyrene degradation in monocultures and co-culture of *A. chroococcum* HN and *P. aminovorans* HPD-2. **b.** Scanning electron micrographs of biofilm in monocultures and co-culture. **c**. EDS analysis of the bridge-like structure observed in the co-culture biofilm of *A. chroococcum* HN and *P. aminovorans* HPD-2. **d.** Observation of pyrene distribution by TP-CLSM. Green fluorescence represents the presence of pyrene on the biofilm of monoculture and co-culture of *A. chroococcum* HN and *P. aminovorans* HPD-2. Error bars represent standard deviations (N = 3).

**Fig. S8** Growth curves of monocultures and co-culture of *A. chroococcum* HN and *P. aminovorans* HPD-2 in nitrogen-free medium with and without the addition of pyrene. Error bars represent standard deviations (N = 3).

**Tables**

**Table 1** Nitrogenase activity of *A. chroococcum* HN and *P. aminovorans* HPD-2 incubated for 24 h by acetylene reduction assay.

**Table 2** Summary of transcriptome sequencing data and transcriptome assembly.

**Table 3** Degradation rates of pyrene on days 1, 2, 3, 5, 7 and 10 of incubation in monocultures and co-culture of *A. chroococcum* HN and *P. aminovorans* HPD-2.

**Table 4** Characterization of pyrene degradation intermediates detected by GC-MS in monocultures and co-culture of *A. chroococcum* HN and *P. aminovorans* HPD-2.

**Table 5** Nitrogen metabolism, aromatic compounds degradation, cell growth and death and TCA cycle related upregulategd gene expression of *A. chroococcum* HN and *P. aminovorans* HPD-2 in co-culture.

**Table 6** Amino acid metabolism related upregulated gene expression of A. chroococcum HN and P. aminovorans HPD-2 in co-culture.

**Table 7** Membrane transport related upregulated gene expression of *A. chroococcum* HN and *P. aminovorans* HPD-2 in co-culture.

**Table 8** Biofilm formation related upregulated gene expression of *A. chroococcum* HN and *P. aminovorans* HPD-2 in co-culture.

**Table 9** Metabolites abundances of amino acids and peptides in monoculture and co-culture of *A. chroococcum* HN and *P. aminovorans* HPD-2. Four replicates (N = 4) were used for metabolomics analyses.

**Table 10** Metabolites abundances of aromatic compounds in monoculture and co-culture of *A. chroococcum* HN and *P. aminovorans* HPD-2. Four replicates (N = 4) were used for metabolomics analyses.


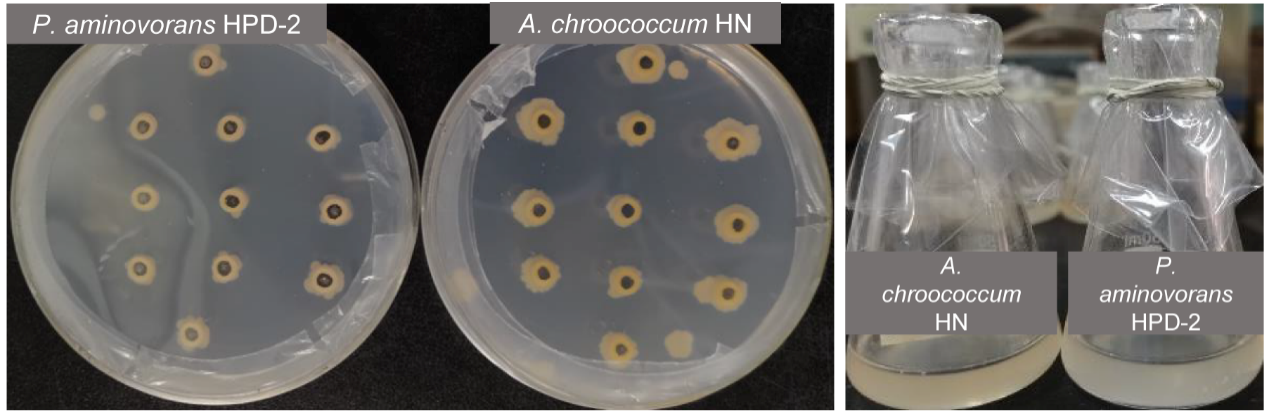


**Fig. S1** Growth of *A. chroococcum* HN and *P. aminovorans* HPD-2 in Brown medium.

**
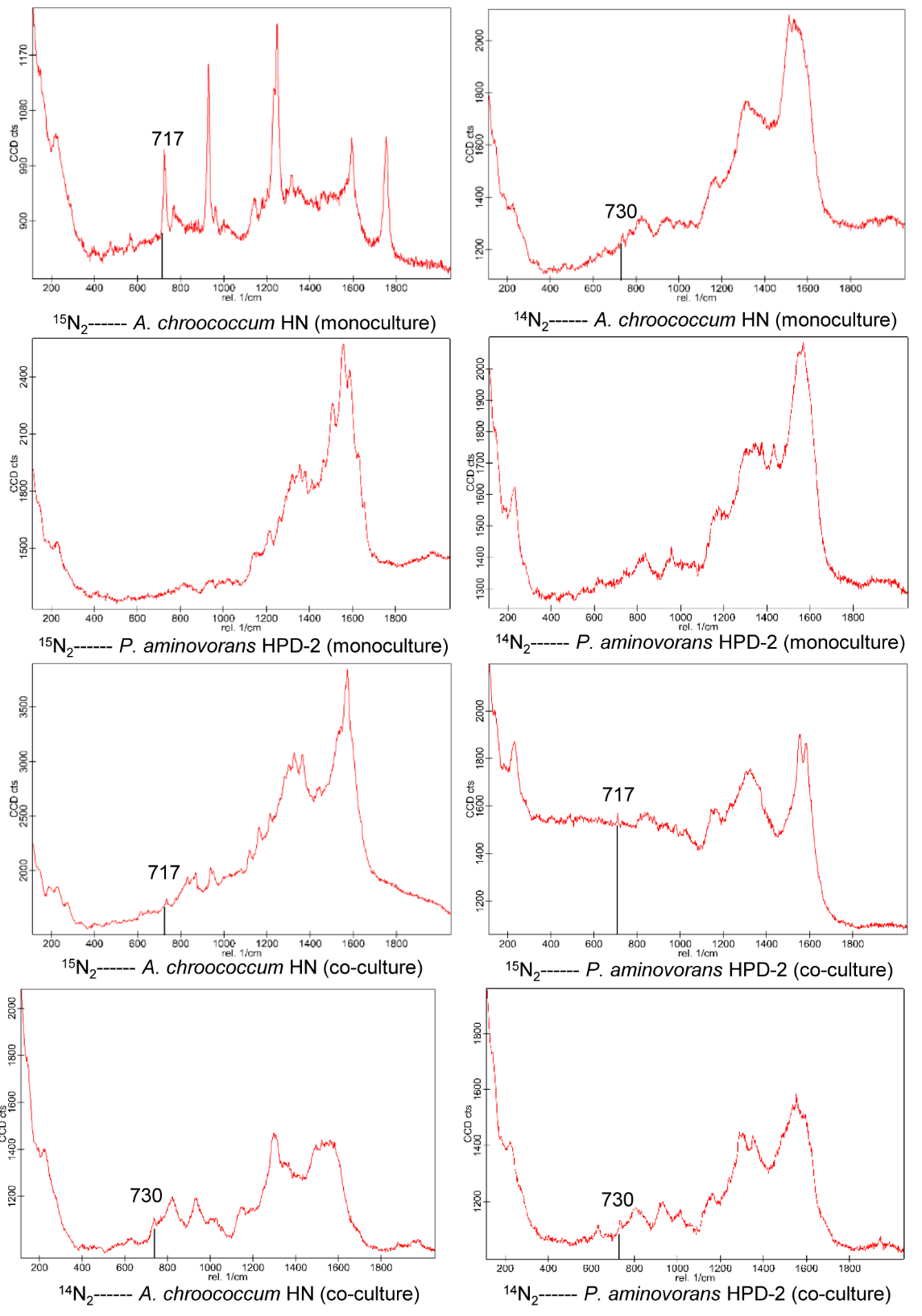
**

**Fig. S2** Uncorrected SERS spectra of *A. chroococcum* HN and *P. aminovorans* HPD-2 incubated with ^15^N_2_ and ^14^N_2_ in monocultures and co-culture.

**
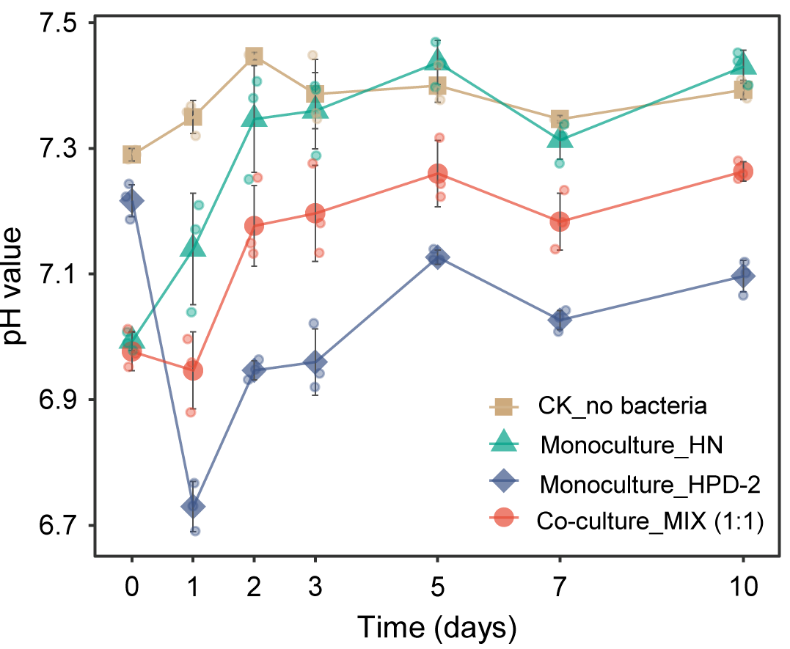
**

**Fig. S3** Change in pH value during pyrene degradation in monocultures and co-culture of *A. chroococcum* HN and *P. aminovorans* HPD-2. Error bars represent standard deviations (N = 3).

**
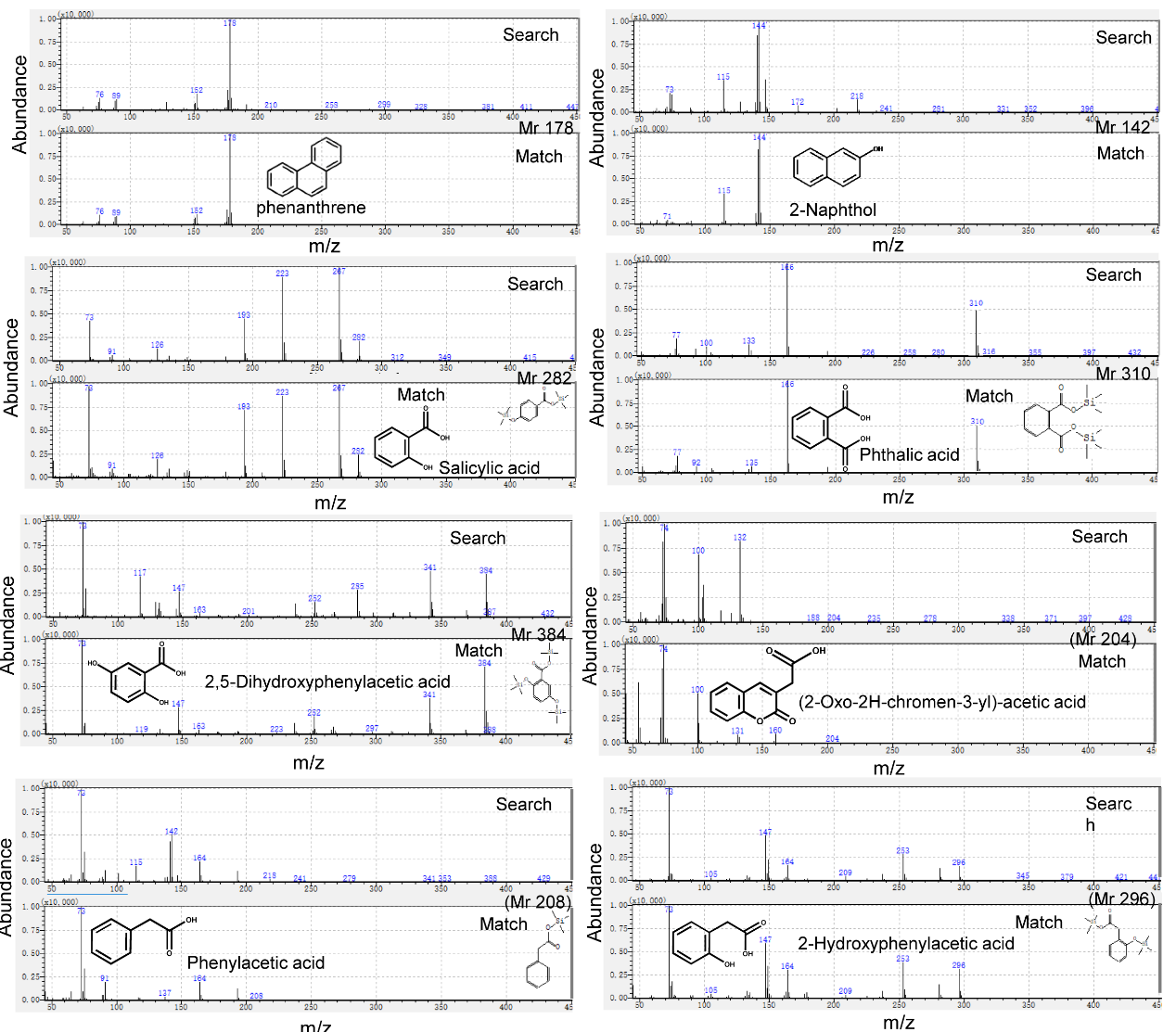
**

**Fig. S4** Mass spectra of the identified metabolites during pyrene degradation in monocultures and co-culture of *A. chroococcum* HN and *P. aminovorans* HPD-2.

**
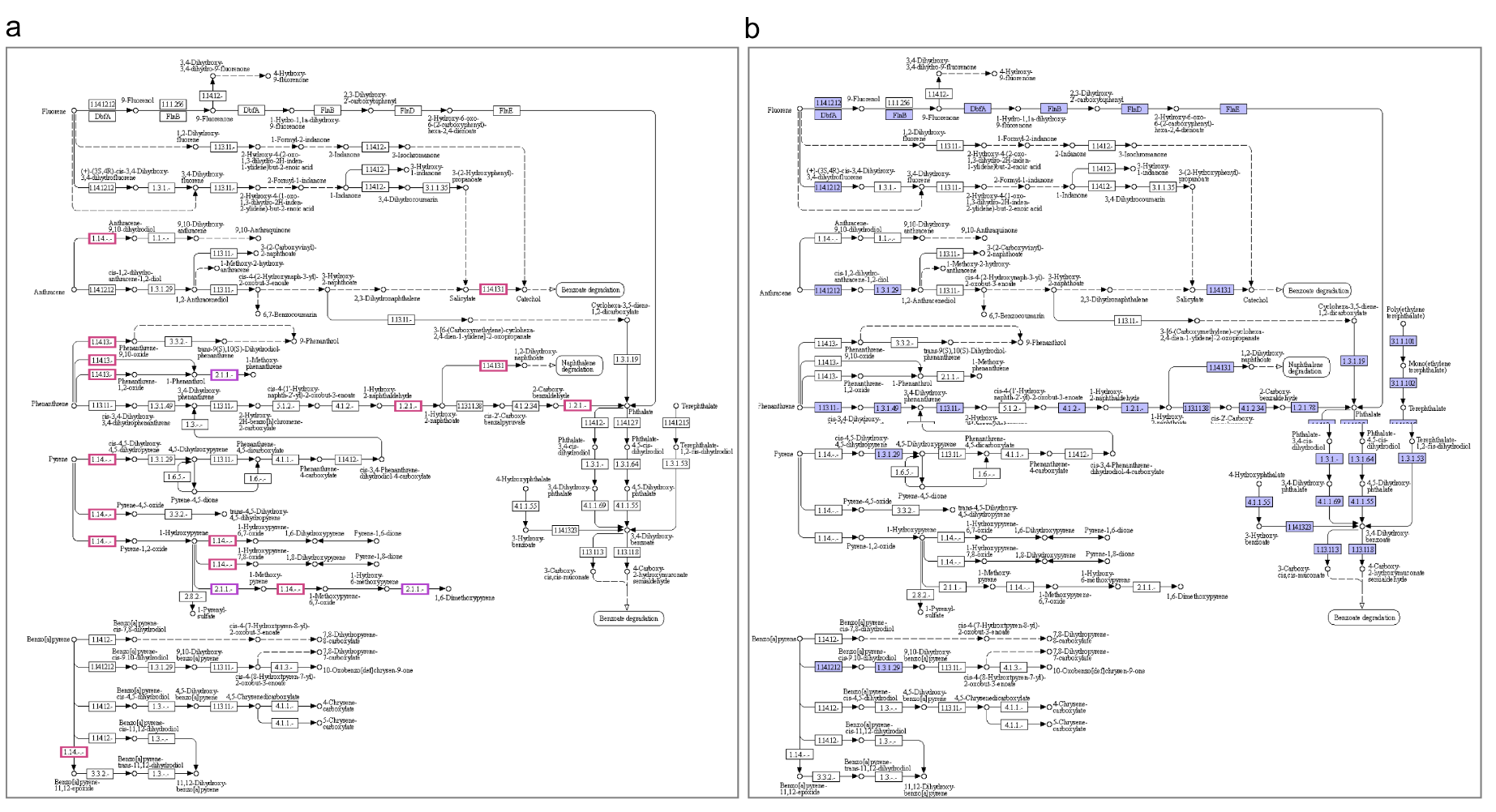
**

**Fig. S5** Functional genes associated with the metabolism of PAHs in the whole genomes of *A. chroococcum* HN (a) and *P. aminovorans* HPD-2 (b). Information on *A. chroococcum* HN was obtained through RAST Server annotation of raw sequence from the National Center for Biotechnology Information. The purple, pink and blue boxes indicate that the enzymes encoded by the genes in the *P. aminovorans* HPD-2 and *A. chroococcum* HN genomes share some similarities with enzymes in metabolic pathways.

**
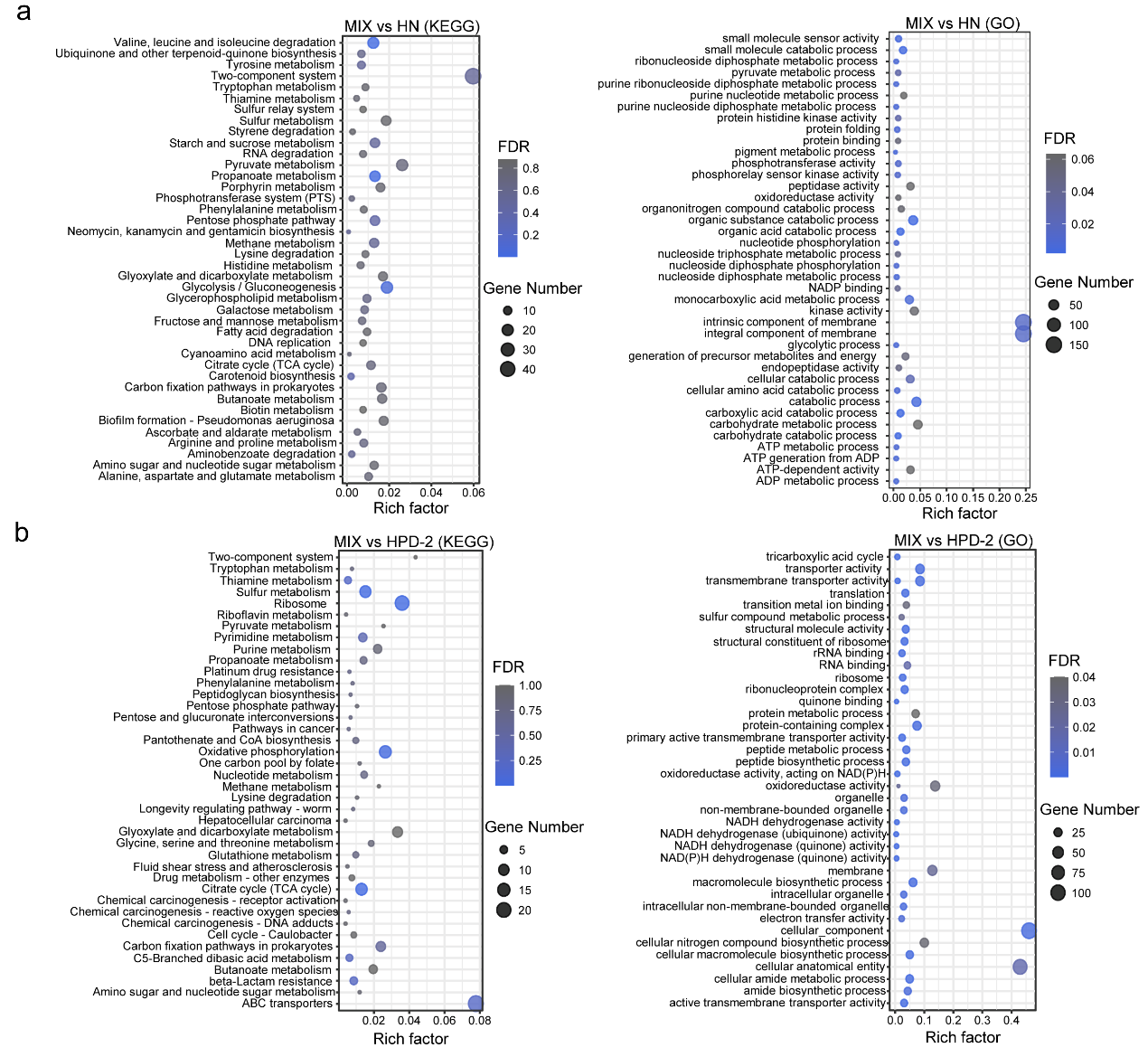
**

**Fig. S6 a.** Analysis of KEEG and GO functional categories down-regulation for *A. chroococcum* HN in the co-culture versus its monoculture. **b.** Analysis of KEEG and GO functional categories down-regulation for *P. aminovorans* HPD-2 in the co-culture versus its monoculture.

**
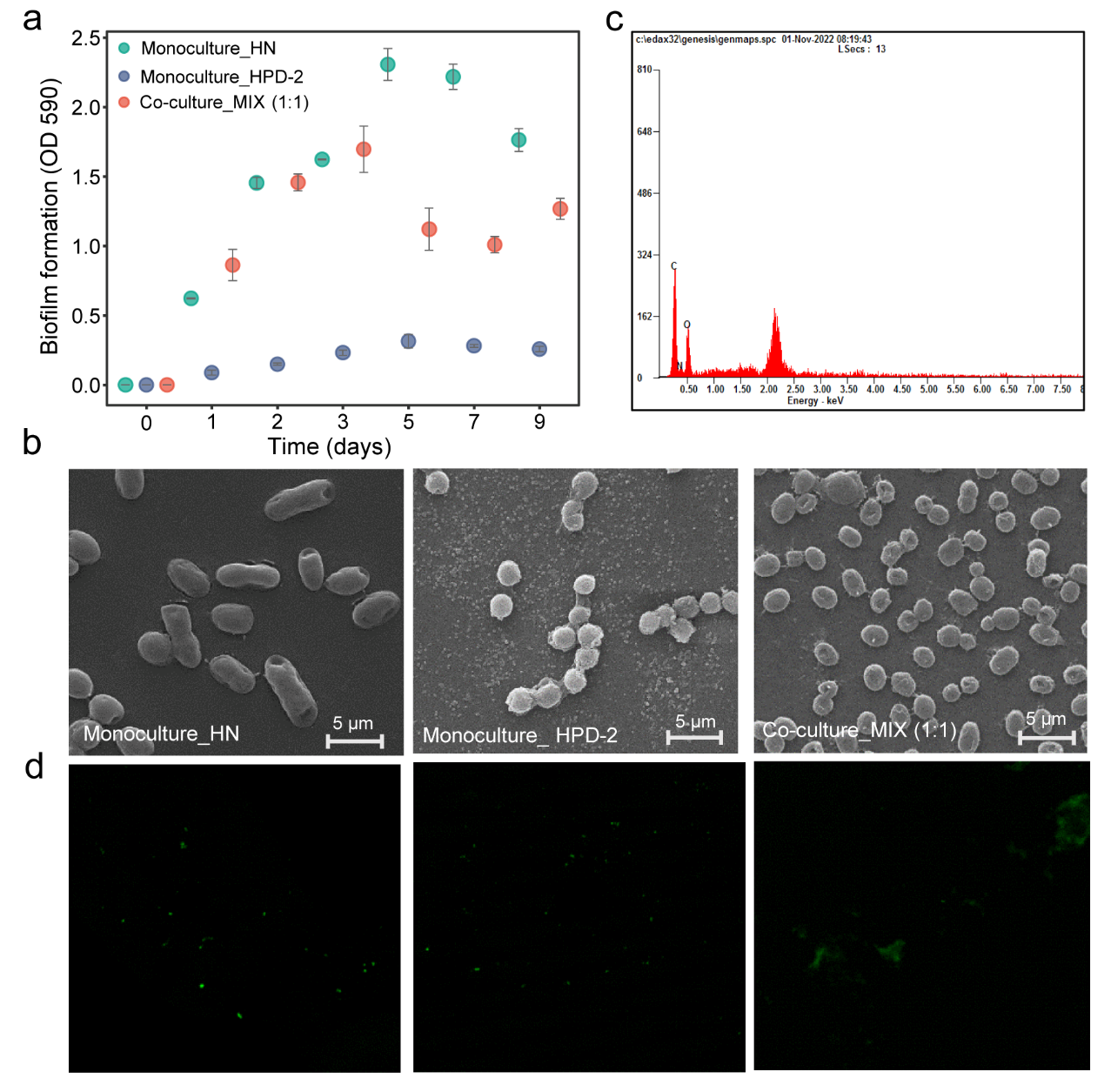
**

**Fig. S7 a.** Change in biomass of biofilms during pyrene degradation in monocultures and co-culture of *A. chroococcum* HN and *P. aminovorans* HPD-2. **b.** Scanning electron micrographs of biofilm in monoculture and co-culture. **c**. EDS analysis of the bridge-like structure observed in the co-culture biofilm of *A. chroococcum* HN and *P. aminovorans* HPD-2. **d.** Observation of pyrene distribution by TP-CLSM. Green fluorescence represents the presence of pyrene on the biofilm of monoculture and co-culture of *A. chroococcum* HN and *P. aminovorans* HPD-2. Error bars represent standard deviations (N = 3).

**
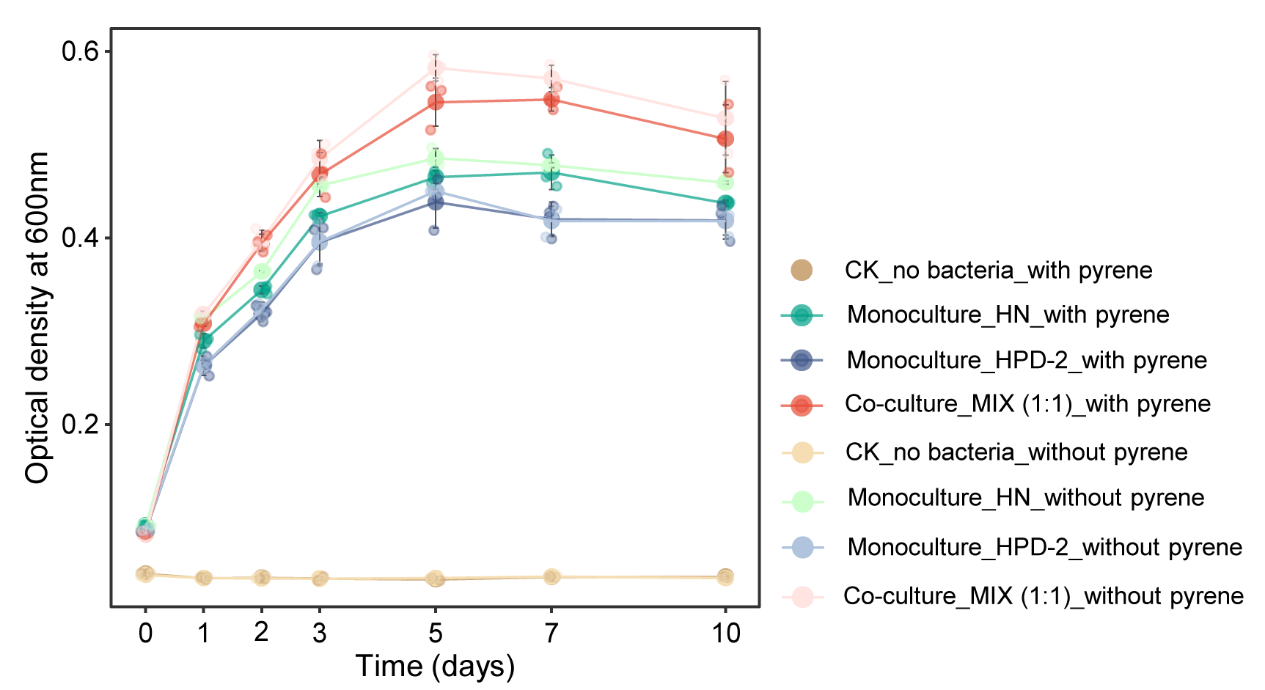
**

**Fig. S8** Growth curves of monocultures and co-cultures of *A. chroococcum* HN and *P. aminovorans* HPD-2 in nitrogen-free medium with and without the addition of pyrene. Error bars represent standard deviations (N = 3).

**Table 1** Nitrogenase activity of *A. chroococcum* HN and *P. aminovorans* HPD-2 incubated for 24 h by acetylene reduction assay.

| **Strain** | **Peak area**  **of ethylene** | **Incubation time**  **(h)** | **Nitrogenase activity (nmol/ml/h)** |
| --- | --- | --- | --- |
| *P. aminovorans* HPD-2 | 2239.1 | 24 | 0.00021 |
| *P. aminovorans* HPD-2 | 2034.2 | 24 | 0.00019 |
| *P. aminovorans* HPD-2 | 1989.8 | 24 | 0.00018 |
| *A. chroococcum* HN | 23600218.9 | 24 | 2.17 |
| *A. chroococcum* HN | 12895073.4 | 24 | 1.19 |
| *A. chroococcum* HN | 16489363.8 | 24 | 1.52 |
| CK | 2133.4 | 24 | 0.00020 |
| CK | 1909.6 | 24 | 0.00018 |
| CK | 1969.5 | 24 | 0.00018 |

CK presents no biological control. All treatments were conducted in triplicates (N = 3).

**Table 2** Summary of transcriptome sequencing data and transcriptome assembly.

| Sample Name | Raw reads | Raw Q20 (%) | Raw Q30 (%) | Clean Reads | Clean Q20 (%) | Clean Q30 (%) |
| --- | --- | --- | --- | --- | --- | --- |
| co-cultures | 29706920 | 97.44 | 94.32 | 29169150 | 98.72 | 96.23 |
| co-cultures | 26906446 | 97.65 | 94.32 | 26625756 | 98.51 | 95.63 |
| co-cultures | 38212772 | 97.72 | 94.88 | 37570272 | 98.81 | 96.5 |
| *P. aminovorans* HPD-2 | 24103662 | 97.25 | 93.87 | 23612736 | 98.36 | 95.25 |
| *P. aminovorans* HPD-2 | 26692906 | 96.02 | 92.51 | 25328472 | 98.4 | 95.44 |
| *P. aminovorans* HPD-2 | 29526882 | 98.01 | 94.89 | 29061858 | 98.6 | 95.71 |
| *A. chroococcum* HN | 27695200 | 95.56 | 90.45 | 27007262 | 97.14 | 92.8 |
| *A. chroococcum* HN | 37204608 | 93.26 | 87.12 | 34866440 | 96.15 | 91.29 |
| *A. chroococcum* HN | 26687926 | 94.6 | 89.08 | 25617470 | 96.85 | 92.29 |

**Table 3** Degradation rates of pyrene on days 1, 2, 3, 5, 7, and 10 of incubation in monocultures and co-culture of *A. chroococcum* HN and *P. aminovorans* HPD-2.

|  | Culture time (days) | | | | | |
| --- | --- | --- | --- | --- | --- | --- |
| Treatments | 1 | 2 | 3 | 5 | 7 | 10 |
| *P. aminovorans* HPD-2 | 10.45 ± 0.03 | 18.76 ± 0.04 | 21.34 ± 0.03 | 24.06 ± 0.04 | 29.01 ± 0.03 | 44.96 ± 0.04 |
| *A. chroococcum* HN | 11.14 ± 0.02 | 13.52 ± 0.04 | 16.67 ± 0.02 | 21.94 ± 0.01 | 24.38 ± 0.02 | 36.36 ± 0.03 |
| *Co-culture* | 14.05 ± 0.05 | 25.92 ± 0.08 | 27.99 ± 0.04 | 32.15 ± 0.04 | 41.47 ± 0.02 | 53.30 ± 0.03 |
| CK | 4.56 ± 0.04 | 9.51 ± 0.06 | 10.02 ± 0.04 | 15.99 ± 0.06 | 19.14 ± 0.05 | 20.91 ± 0.03 |

Results are in the form mean ± standard deviation, N = 3.

**Table 4** Characterization of pyrene degradation intermediates detected by GC-MS in monocultures and co-culture of *A. chroococcum* HN and *P. aminovorans* HPD-2.

| ***A. chroococcum* HN** | | ***P. aminovorans* HPD-2** | | **Co-culture** | |
| --- | --- | --- | --- | --- | --- |
| **Time** | **Name of metabolites** | **Time** | **Name of metabolites** | **Time** | **Name of metabolites** |
| 12.372/12.017 | 2-Naphthol | 11.729 | (2-Oxo-2H-  chromen-3-yl)-acetic acid | 12.024 | 2-Naphthol |
| 19.815 | Salicylic acid | 11.884 | Phenylacetic acid | 15.660 | Phthalic acid |
| 23.023 | phenanthrene | 18.522 | 2-Hydroxy  phenylacetic acid | 21.125 | 2-Hydroxyphenylacetic acid |
| 29.627 | pyrene | 19.817 | Salicylic acid | 23.019 | phenanthrene |
|  |  | 21.115 | 2,5-Dihydroxyphenylacetic acid  phenanthrene | 29.625 | pyrene |
|  |  | 23.025 | phenanthrene |  |  |
|  |  | 29.620 | pyrene |  |  |
